# The Spatial Relationship and Surface Projection of Canine Sciatic Nerve and Sacrotuberous Ligament: A Perineal Hernia Repair Perspective

**DOI:** 10.1371/journal.pone.0152078

**Published:** 2016-03-22

**Authors:** Nabin Khatri-Chhetri, Rupak Khatri-Chhetri, Cheng-Shu Chung, Rey-Shyong Chern, Chi-Hsien Chien

**Affiliations:** 1 Department of Veterinary Medicine, College of Veterinary Medicine, National Pingtung University of Science and Technology, Pingtung, Taiwan; 2 Department of Small Animal Surgery and Acupuncture, Animal Hospital, National Pingtung University of Science and Technology, Pingtung, Taiwan; 3 Department of Veterinary Anatomy, College of Veterinary Medicine, National Pingtung University of Science and Technology, Pingtung, Taiwan; University of Bari, ITALY

## Abstract

Sciatic nerve entrapment can occur as post-operative complication of perineal hernia repair when sacrotuberous ligament is incorporated during hernia deficit closure. This results in sciatic sensory loss and paralysis of the hind leg. This study investigated the spatial relationship of sciatic nerve and sacrotuberous ligament and their surface topographic projection of 68 cadavers (29 Beagles and 39 Taiwanese mongrels) with various heights (25–56 cm). By gross dissection, the sacrotuberous ligament and sciatic nerve were exposed and their distance in between was measured along four parts (A, B, C, D) of sacrotuberous ligament. The present study revealed that the C was the section of sacrotuberous ligament where the sciatic nerve and the sacrotuberous ligament are closest to each other. Furthermore, a positive correlation was observed between C and height of the dogs. From the present study, we found that the C in smaller dogs has the shortest distance between the sciatic nerve and the sacrotuberous ligament, and thus the most vulnerable to sciatic nerve entrapment, and needs to be avoided or approached cautiously during perineal hernia repair.

## Introduction

Perineal hernia is a common disease condition in dogs resulting from a weakness or a separation, and eventually a failure of the pelvic diaphragm muscles, which results in herniation of the pelvic or abdominal contents into the subcutaneous perineum [1–3]. The urinary bladders, the prostrate, mesenteric fats, parts of the small intestines, and the uterine body have been reported to be herniated in the perineal sac [1], making this condition extremely problematic. Medical or conservative therapy is unlikely to solve the problem permanently and requires herniated content repositioning and reconstruction of the weak pelvic diaphragm [4].

Various herniorrhaphy techniques have been used alone or in combination with coloplexy or cystoplexy depending on the type of herniation [5]. The most common techniques used include the traditional or anatomic re-apposition and the flap techniques [6–10]. Due to perineal diaphragm muscles atrophy in majority of cases, simple apposition of the coccygeus, levator ani, and external anal sphincter muscles may not be possible or may predispose to recurrence [9, 11]. Hence, sacrotuberous ligament is often incorporated as necessary to close ventral and lateral part of the hernial deficit [3, 4]. Sciatic nerve paralysis and sciatic nerve neuropraxia are often reported as a post-operative complication when sacrotuberous ligament is incorporated in the suture bite [1, 12–15].

Sacrotuberous ligament is a fibrous cord that is flattened at both of its ends and extends from the caudo-lateral part of the apex of the sacrum and the transverse process of the first caudal vertebra to the lateral angle of the ischiatic tuberosity [16]. Sciatic nerve runs dorsolateral to the sacrotuberous ligament, exiting the pelvis towards the thigh [17]. Most of the available literatures have reported the close association of sacrotuberous ligament and sciatic nerve and vulnerability of sciatic nerve entrapment in perineal hernia repair. However, proximity and spatial relationship of sciatic nerve and sacrotuberous ligament have not been reported. In the present study, we described the spatial relationship of sciatic nerve along the sacrotuberous ligament, which in our opinion gives the surgeon invaluable information to avoid sciatic entrapment and the associated complications during surgical repair of a perineal hernia.

## Materials and Methods

### Ethics statement

All the cadavers used in the present study were the anatomical specimens from the archival of Department of Veterinary Anatomy, National Pingtung University of Science and Technology (NPUST). None of the dogs were sacrificed purposefully for this research. The approval was obtained from the Department of Veterinary Anatomy for using the dog carcasses for the measurement. Additional approval from the ethics committee was not required for using these cadavers.

### Cadavers

Sixty-eight canine cadavers were obtained from the Department of Veterinary Anatomy, College of Veterinary Medicine, NPUST. Out of 68 Cadavers, 29 were purebred Beagles and 39 were Taiwanese mongrels with various heights. All the dogs used in the present study were adults and were at least 2 years old. The dogs were classified into three categories on the basis of their height at withers: small (20–40 cm), medium (40–50 cm) and large (50–60 cm).

### Proximity of sacrotuberous ligament and sciatic nerve

**Fig 1:**
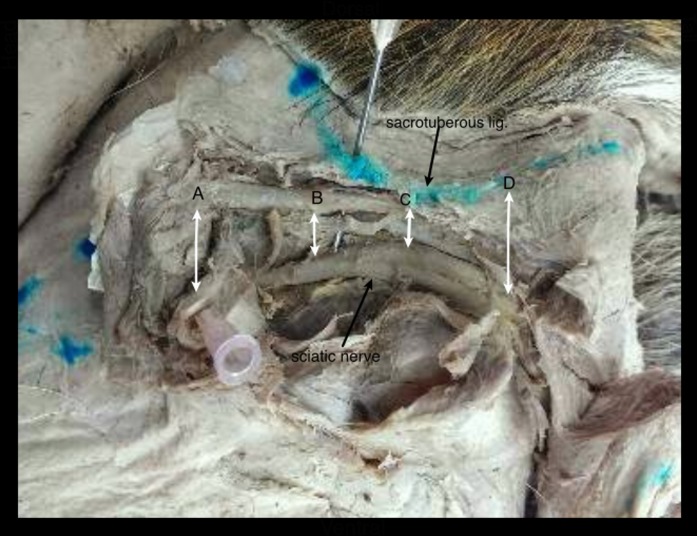
Well-exposed view of sciatic nerve and sacrotuberous ligament in perineal hernia in a dog: A) Proximal attachment of the sacrotuberous ligament; B) 1/3^rd^ distance from the proximal attachment; C) 2/3^rd^ distance from the proximal attachment; and D) Distal attachment of the sacrotuberous ligament.

Both gross and micro dissection technique were used to expose the sciatic nerve and the sacrotuberous ligament. The sacrotuberous ligament was fully exposed along the full length of the sacrotuberous ligament. Sacrotuberous ligament was divided into four equal parts: (A) proximal attachment of the sacrotuberous ligament at the level of apex of the sacrum and the transverse process of first caudal vertebra; (B) 1/3^rd^ distance from the proximal attachment; (C) 2/3^rd^ distance from the proximal attachment; and (D) distal attachment at the level of lateral angle of the ischiatic tuberosity (Fig 1). These distances were pinpointed for accurate calibration. Following these, distance of the sciatic nerve from each point (A, B, C & D) of the sacrotuberous ligament was also measured. All the measurements were taken by two veterinary surgeons. Both surgeons decided unanimously on the appropriate measurement site for each parameter and concurred on the accuracy of the measurements. All measurements were precisely hand calibrated (0.1 cm), thus enabling the identification of easily recognizable points.

### Statistical analysis

All statistical analyses were performed using IBM SPSS Statistics for Windows, version 21.0 (IBM Corp., Armonk, NY, USA), mean, standard deviation, and range were calculated for each parameter. The degree of linear relationship between two variables was analyzed using Pearson’s correlation coefficient. One-way ANOVA followed by Tukey’s or Games Howell test was performed to test the differences between the means. Difference with *P*<0.05 was considered significant. Graphs were prepared using SigmaPlot version 10.0 (Systat Software, Inc., San Jose California, USA).

## Results

### The Cadaver groups

Beagles were the smallest of the entire cadaver group, and the height at the wither ranged from 25 to 56 cm. All Beagles belonged to small dog group while the mongrel’s height ranged from 27 to 56 cm at withers, and were further subcategorized into small (n = 11), medium (n = 18) and large (n = 10). In the present study, the smallest mongrel measured was 27 cm at wither whereas the tallest dog measured was 56 cm at wither.

### Sciatic and sacrotuberous ligament proximity

All the dogs included in the present study had sacrotuberous ligament attached to the apex of sacrum and the transverse process of first caudal vertebra cranially and to the lateral angel of the ischiatic tuberosity caudally. The sciatic nerve exited the pelvis through the greater ischiatic notch and ran caudally towards the hip joint, caudal to the greater trochanter. The course of the sciatic nerve was found to be curvilinear in perspective to sacrotuberous ligament.

In beagles, the closest distance of sciatic nerve to sacrotuberous ligament was from C and the least measured distance at that level was 0.3 cm (0.57±0.15 cm). The longest distance of sciatic nerve was measured from A (1.16±0.69 cm) and ranged from 0.9 to 1.5 cm. The sciatic nerve distance from point C of sacrotuberous ligament was the shortest, and was significantly shorter (*P<0*.*05*) from A, B and D while A, D was the farthest (*P<0*.*05*) (Table 1).

**Table 1:** Proximity distance of sciatic nerve and sacrotuberous ligament along various points A, B, C, and D in Beagles.

			Distances between sciatic nerve and sacrotuberous ligament (cm)							
	Heights (cm)		A		B		C		D	
	Mean±SD	Range	Mean±SD	Range	Mean±SD	Range	Mean±SD	Range	Mean±SD	Range
**All dogs (n = 68)**	38.64±8.67	25–56	1.42±0.39^a^	0.9–2.6	0.97±0.35^b^	0.4–1.9	0.7±0.29^c^	0.3–1.5	1.24±0.49^a^	0.5–3
**Beagles**										
Small (20–40) (n = 29)	31.43±3.5	25–38.2	1.16±0.69^a^	0.9–1.5	0.77±1.88^b^	0.5–1.2	0.57±0.15^c^	0.3–0.9	1.1±0.26^a^	0.6–1.5

(A) Proximal attachment of the sacrotuberous ligament at the level of apex of the sacrum and the transverse process of first caudal vertebra; (B) 1/3rd distance from the proximal attachment; (C) 2/3rd distance from the proximal attachment; and (D) distal attachment at the level of lateral angle of the ischiatic tuberosity

SD = standard deviation; n = total number of cadaver; A, B, C and D are divisions of the sacrotuberous ligament.

Values bearing different letters are significantly different from one another (P<0.05) as determined by one-way ANOVA and followed by Tukey’s post hoc analysis.

In small mongrel group (20–40 cm), the closest distance of sciatic nerve to sacrotuberous ligament was from C and measured 0.3 cm. Longest sciatic distance measured was from A (1.28±0.29 cm). The sciatic nerve distance from point C of sacrotuberous ligament was the shortest and was significantly shorter (*P<0*.*05*) from A and D (Table 2).

**Table 2:** Proximity distance of sciatic nerve and sacrotuberous ligament along various points A, B, C, and D in mongrels.

			Distances between sciatic nerve and sacrotuberous ligament (cm)							
	Heights (cm)		A		B		C		D	
	Mean±SD	Range	Mean±SD	Range	Mean±SD	Range	Mean±SD	Range	Mean±SD	Range
**All dogs (n = 68)**	38.64±8.67	25–56	1.42±0.39^a^	0.9–2.6	0.97±0.35^b^	0.4–1.9	0.7±0.29^c^	0.3–1.5	1.24±0.49^a^	0.5–3
**Mixed**										
All (n = 39)	44±7.36	27–56	1.61±0.4^a^	0.9–2.6	1.16±0.36^b^	0.5–1.9	0.70±0.28^c^	0.3–1.4	1.37±0.57^ab^	0.7–3
Small (20–40) (n = 11)	34.54±5.14	27–40.7	1.28±0.29^a^	0.9–2	0.88±0.4^bc^	0.5–1.9	0.55±0.19^bc^	0.3–0.8	1.03±0.24^a^	0.7–1.5
Medium (40–50) (n = 18)	45.35±2.46	41.5–49.5	1.67±0.29^a^	1.1–2.1	1.23±0.27^b^	0.7–1.8	0.83±0.19^c^	0.5–1.2	1.34±0.46^a^	0.7–2
Large (50–60) (n = 10)	52.02±1.96	50–56	1.85±0.47^a^	1.1–2.6	1.34±0.32^bc^	0.8–1.9	0.98±0.33^bc^	0.5–1.4	1.79±0.76^a^	0.9–3

(A) Proximal attachment of the sacrotuberous ligament at the level of apex of the sacrum and the transverse process of first caudal vertebra; (B) 1/3rd distance from the proximal attachment; (C) 2/3rd distance from the proximal attachment; and (D) distal attachment at the level of lateral angle of the ischiatic tuberosity

SD = standard deviation; n = total number of cadaver; A, B, C and D are divisions of the sacrotuberous ligament.

Values bearing different letters are significantly different from one another (P<0.05) as determined by one-way ANOVA followed by Tukey’s or Games Howell post hoc analysis.

In the medium sized mongrel group (40–50 cm), the shortest distance of sciatic nerve to sacrotuberous ligament was from C and measured 0.5 cm. Sciatic distance from A (1.67±0.29 cm) was significantly (*P<0*.*05*) longer than A, B and C. The sciatic nerve distance from C of the sacrotuberous ligament was the shortest and was significantly shorter than A, B and D (P<0.05) (Table 2).

In the large sized mongrel (50–60 cm), the shortest distance of sciatic nerve to sacrotuberous ligament measured 0.5 cm. Sciatic distance from A (0.85±0.47 cm) was the farthest. The sciatic nerve distance from C of sacrotuberous ligament was the shortest, and was significantly shorter than A and D (*P<0*.*05*) (Table 2).

**Fig 2:**
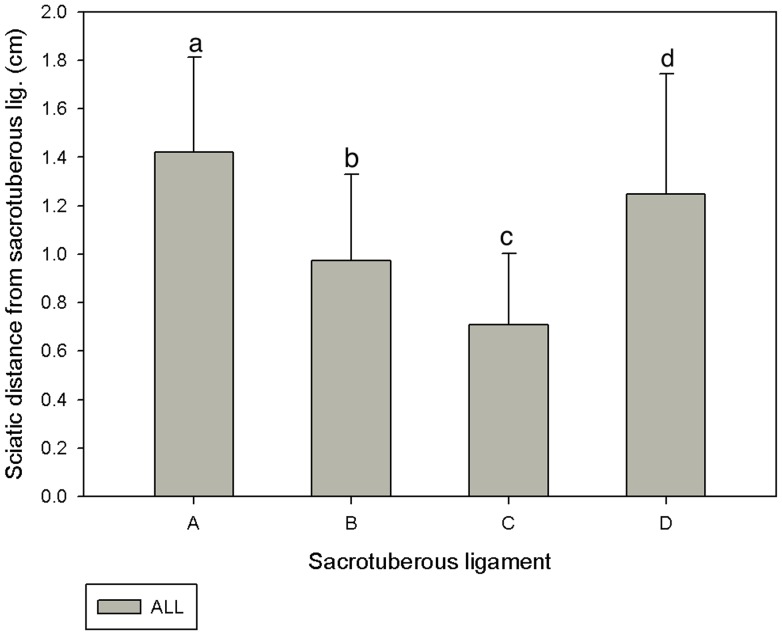
Sciatic nerve distances from different divisions of the sacrotuberous ligament A, B, C and D in 68 cadavers. Results are expressed as mean±SD. Letters above the bar indicate that these sections differ significantly from each other (a, b, c: *P*<0.05).

**Fig 3:**
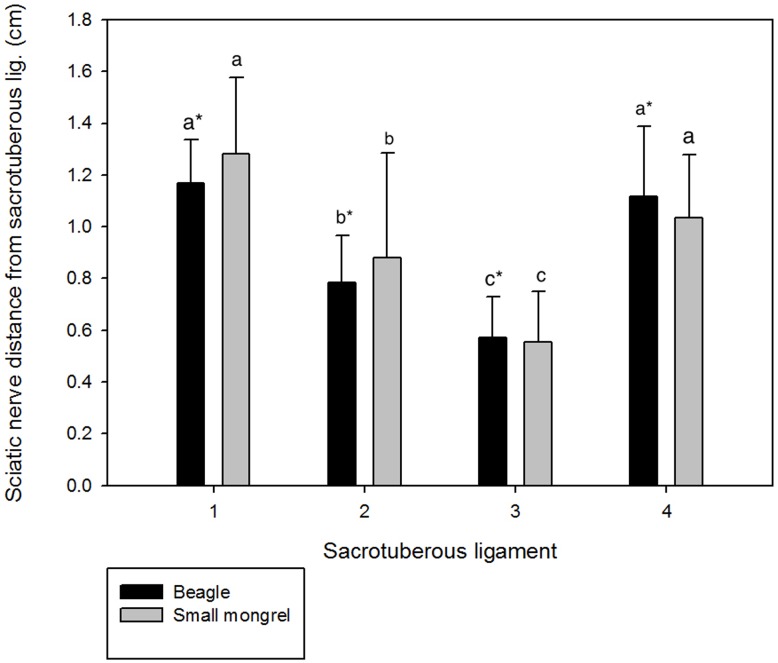
Sciatic nerve distances from different divisions of the sacrotuberous ligament A, B, C and D in Beagles and mongrels. **Results are expressed as mean±SD.** Letters above the bar indicate that these sections differ significantly from each other (a, b, c: *P*<0.05); * indicates the significance between Beagles and mongrels *(P*<0.05).

**Fig 4:**
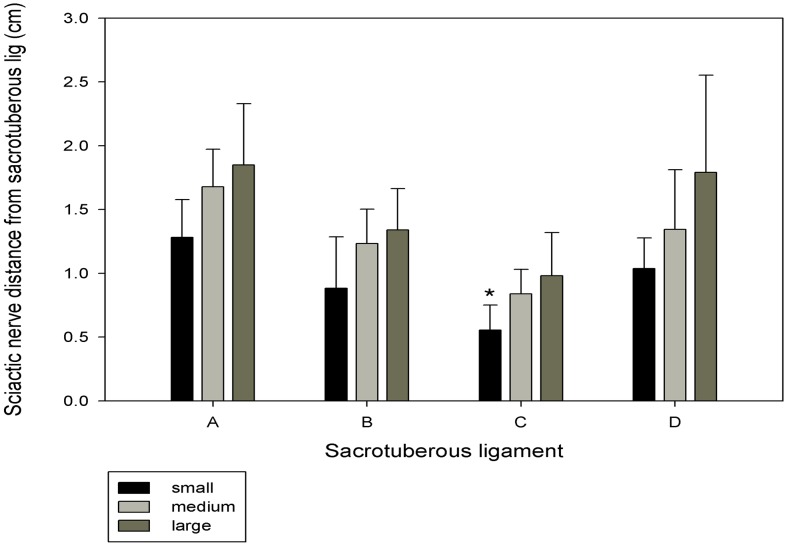
Sciatic nerve distance from different divisions of the sacrotuberous ligament A, B, C and D in small, medium, and large mongrel groups. Results are expressed as mean±SD. Letters above the bar indicate that these sections differ significantly from each other (*: *P*<0.05).

In all the dogs, the distance closest to widest sciatic nerve from sacrotuberous ligament was C, B, D and A respectively (Fig 2). The sciatic nerve distances from point A, B, C, and D were found to be significantly different (*P<0*.*05*) between the Beagle and the mongrel groups (Fig 3). Similarly, the sciatic nerve distance from C of small group was found to be significantly shorter (*P<0*.*05*) compared to other groups (Fig 4).

**Fig 5:**
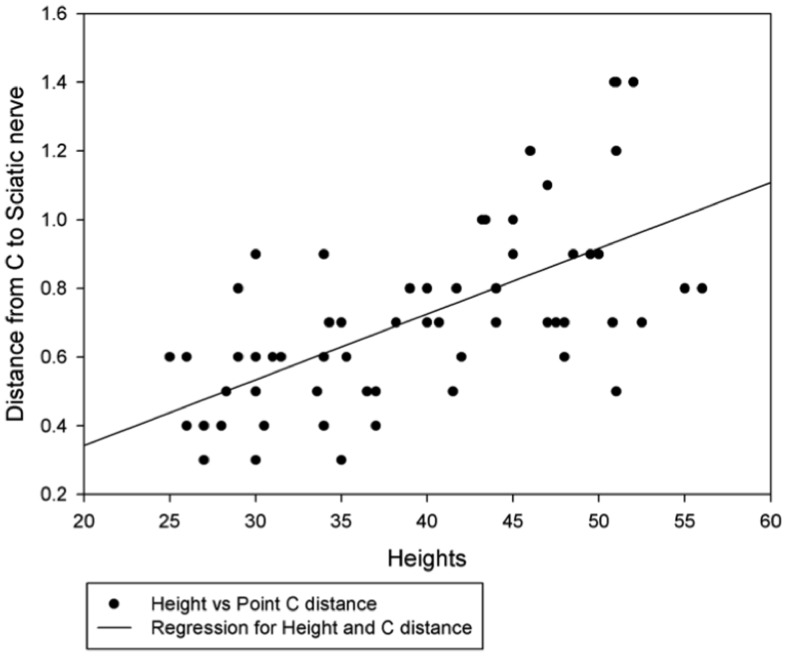
Correlation and regression between height and distance of the sciatic nerve from C of sacrotuberous ligament. **r = 0.64, *P*<0.01.** Effect size is larger or larger than the typical.

Pearson correlation analysis between the sciatic nerve distances from point A, B, C and D with the height of the dogs (Table 3) showed significant positive correlations between distances from C and sciatic nerve (r = 63, P<0.01; Fig 5). Similarly, positive correlation were observed between: heights, and distance of A and sciatic nerve (r = 0.71, P<0.01, Table 3); heights, and distance of B and sciatic nerve (r = 69, P<0.01, Table 3); and heights and distance of D and sciatic nerve (r = 0.37, P<0.05, Table 3). The sciatic nerve distance increased with the increase in the height of the dog.

**Table 3:** Correlation between height and different sections of the sacrotuberous ligament A, B, C, and D.

		Height	A	B	C	D
**Height**	Pearson Correlation	1	.710**	.698**	.635**	.378**
**Height**	Sig. (2-tailed)		.000	.000	.000	.002
**Height**	N	68	68	68	68	68
**A**	Pearson Correlation	.710**	1	.866**	.815**	.561**
**A**	Sig. (2-tailed)	.000		.000	.000	.000
**A**	N	68	68	68	68	68
**B**	Pearson Correlation	.698**	.866**	1	.852**	.526**
**B**	Sig. (2-tailed)	.000	.000		.000	.000
**B**	N	68	68	68	68	68
**C**	Pearson Correlation	.635**	.815**	.852**	1	.625**
**C**	Sig. (2-tailed)	.000	.000	.000		.000
**C**	N	68	68	68	68	68
**D**	Pearson Correlation	.378**	.561**	.526**	.625**	1
D	Sig. (2-tailed)	.002	.000	.000	.000	
D	N	68	68	68	68	68

(A) Proximal attachment of the sacrotuberous ligament at the level of apex of the sacrum and the transverse process of first caudal vertebra; (B) 1/3rd distance from the proximal attachment; (C) 2/3rd distance from the proximal attachment; and (D) distal attachment at the level of lateral angle of the ischiatic tuberosity

** Correlation is significant at the 0.01 level (2-tailed)

A, B, C, and D are divisions of the sacrotuberous ligament.

## Discussion

### Perineal hernia and sciatic nerve paralysis

Perineal hernia is a common problem that mostly affects middle-aged to older or intact male dogs [14]. Several techniques with overall good to excellent outcomes have been used to surgically correct perineal hernia. In a retrospective study done by Weaver and Omamegbe (1981), standard apposition technique has been shown to have a higher success rate (81%) than the superficial gluteal flap technique (64%). In addition, recurrence rate has been reported to be higher with the superficial gluteal flap technique (14.3%) compared to the standard technique (8.7%). The ligamentous tissue such as ventral fascia for an umbilical hernia, inguinal ligament and tendinous aponeurosis for inguinal hernia are usually used for repair of abdominal hernias.

In case of perineal hernia repair, the sacrotuberous ligament is the only structure with the strength similar to that of the ventral fascia, the inguinal ligament, and the tendinous aponeurosis [12]. The sacrotuberous ligament used for apposition in the traditional apposition technique might be the reason for increased strength of the deficit. Hence, less recurrence is observed with this technique in comparison to the flap techniques, which often utilizes the soft tissue to anchor the flap [8, 9, 18]. Therefore, incorporation of sacrotuberous ligament is often important in cases where there are friable tissue or atrophied muscle, so as to increase the strength of perineal diaphragm repair.

Sciatic nerve paralysis is one of the important post-operative complications of perineal hernia repair [1, 7, 12, 13], which is often irreversible, leading to permanent paralysis of the affected legs. Alongside the heavy muscle coverage that limits its visibility, the sciatic nerve is well protected and deeply located close to the pelvic bone, rendering this nerve vulnerable to iatrogenic injuries [12, 13]. The occurrence of sciatic nerve was 5.5%, 3%, and 5% in the studies done by Burrows and Harvey (1973), Weaver and Omamegbe (1981) and Forterre *et al*., 2007, respectively.

The sciatic nerve and the sacrotuberous ligament are in close proximity with each other [16]. Sciatic entrapment usually occurs when sutures are placed around the sacrotuberous ligament [12, 13]. Sciatic neuropraxia, which is a temporary localized conduction blockage, can also occur due to extension of tension on the nerve or ischemia [19, 20]. Furthermore, pressure elicited by fibrotic tissues during the healing process causes compression of the sciatic nerve, resulting in sciatic neuropraxia or paralysis [10]. Most of the current literatures suggest avoiding the sacrotuberous ligament [4], but in case of coccygeus muscle and levator ani muslce atrophy or friablility, the sacrotuberous ligament needs to be incorportaed to increase the tensile strength of the hernia deficit. In our opinion, excellent surgical skills, along with adequate knowledge on the spatial relationship of the sacrotuberous ligament and the sciatic nerve can help avoid sciatic nerve entrapment as a complication during the perineal hernia repair. The surgeon will be able to use the sacrotuberous ligament to increase the tensile strength of the hernial repair without worry of sciatic entrapment.

### Spatial relationship of sciatic nerve and sacrotuberous ligament

In the present study, we found that the course of sciatic nerve in the perineal space is slightly curvilinear and its proximity distance varies along the sacrotuberous ligament. This is because sciatic nerve runs close to the pelvic bone after emerging from the greater ischiatic notch [21]. Along the acetabular ridge, the pelvis floor is slightly elevated near the body of the ischium [22, 23], which elevates the sciatic nerve and contributes to its curvilinear appearance.

In all the dogs measured, the distance of the sciatic nerve from point C was the shortest (*P<0*.*05*). The sciatic nerve at this level was approximately close to the acetabular ridge. In Beagles, the distance of the sciatic nerve from point C was significantly different from A, B and D, whereas the distance of sciatic nerve from point A and D were not significantly different. This is due to the curvilinear course of the sciatic nerve along the sacrotuberous ligament. Similar observation was seen in the medium sized mongrels. In small and large sized mongrel groups, however, the distances of sciatic nerve from B and from C were shorter significantly (p<0.05), but significance of the differences between these two distances was inconclusive. This is probably attributable to the variation among different dog breeds in the pelvic tilt (angulation), which is an angle of the long axis of the dog pelvis from the iliac crest to the most distal point of the ischiatic tuberosity, relatively to the horizon [24]. The sacrotuberous ligament is attached distally to the ischiatic tuberosity. Any changes in the pelvic tilt would change the spatial position of the sacrotuberous ligament within the pelvis, hence changing its proximity with the sciatic nerve. The breed associated variations of the pelvic angulation and changes that it makes to the proximity of sciatic nerve and sacrotuberous ligament are beyond the scope of this study.

Beagles had the shortest distances of sciatic nerve from point A, B, C, and D that were significantly different from the mongrel groups. This is because of the significant height difference between these two groups, as Beagles were significantly shorter than the mongrels in the present study. Furthermore, positive correlation between the heights and the distances of sciatic nerve from A, B, C and D was observed (*P<0*.*05*). Larger and taller dogs have larger and longer bones when compared to the shorter dogs. Their pelvic bones are larger and longer too; their ischiatic tuberosities are wider compared to those of the smaller breeds [25], and variation of pelvic size has been found to be correlated with the animal height [26]. This variation causes the difference in the sacrotuberous ligament length and wider positioning of the attachments and thus, increases the distances from the sciatic nerve. Our study showed that this variation is significantly correlated with the animal height. Another factor that usually contributes to this variation is the genetic trait [27]. However, proximity study of sciatic nerve and sacrotuberous ligament in various breeds was beyond the scope of this study.

### Sciatic nerve and sacrotuberous ligament surface topography

**Fig 6:**
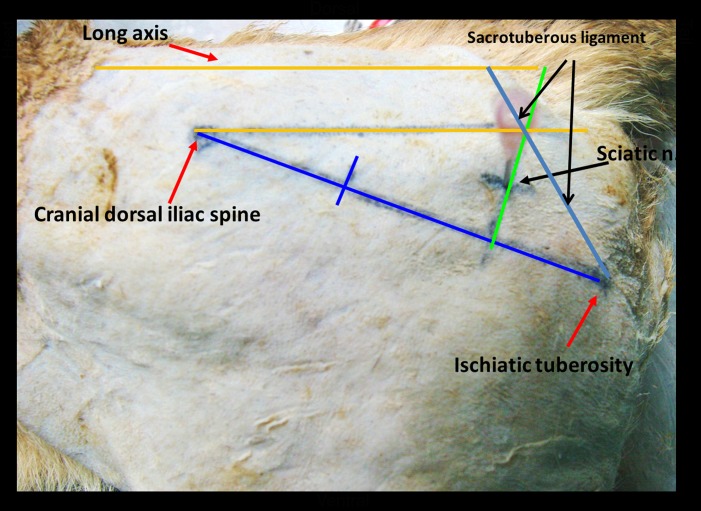
Anatomical landmarks used for locating the left sciatic nerve and the sacrotuberous ligament in a dog. A line has been drawn to connect the cranial dorsal iliac spine and the ischiatic tuberosity. Another line is drawn perpendicular to the distal third of the first line. A third line is drawn from the dorsal iliac spine parallel to the midline, crossing the perpendicular line. The midway of the perpendicular line between the intersection of the cranial dorsal iliac spine and the ischiatic tuberosity is the approximate location of the sciatic nerve (needle). A line drawn from the distal ischiatic tuberosity to the sacrococcygeal joint is the approximate surface projection of the sacrotuberous ligament.

A previous study conducted by Stephan used the dorsal iliac spine and the ischiatic tuberosity as the landmarks and techniques to locate the sciatic nerve [28–30]. In the present study, we used the same landmarks and technique to locate the sciatic nerve. The sacrotuberous ligament can be easily palpated and located by drawing lines form the apex of the sacrum to the lateral angle of the ischiatic tuberosity (Fig 6). Apex of the sacrum is close to the sacro-coccygeal joint and is easily identifiable, whereas the ischiatic tuberosity is a bony prominence that is easily palpable. In addition, the distal sacrotuberous ligament can be palpated from the body surface. This knowledge is useful in approximating the surface projection of the sciatic nerve and sacrotuberous ligament prior to the incision.

## Conclusion

The sacrotuberous ligament is a strong fibrous tissue, which can be utilized in perineal hernia repair. The chances of sciatic nerve entrapment can be minimized by careful selection of the section of sacrotuberous ligament. Our study showed, section C was the closest distance to sacrotuberous ligament, and this distance varies with height of the dog. Thus, height of the dog was considered to be the contributing factor for variation of the distances of sciatic nerve and sacrotuberous ligament. In beagles and smaller dogs, sciatic distances to sacrotuberous ligament are comparatively shorter and point C was the closest to sciatic nerve. Hence, surgeons should be very careful while incorporating this section of sacrotuberous ligament in smaller breeds. This explains the vulnerability of sciatic nerve entrapment in smaller breeds. In larger breeds, distances of sciatic nerve and sacrotuberous ligament are usually larger, which makes sciatic nerve less vulnerable to entrapment. However, since C is the closest distance, we suggest either avoiding this section or approaching cautiously. These findings give invaluable information on spatial relationship and topographic projection of the sciatic nerve and the sacrotuberous ligament, and thus, needs to be considered to help prevent sciatic nerve entrapment during a perineal hernia repair.

Variations in the pelvic angulation and genetic variations of the pelvic size among different breeds are well documented and can cause variations in the distances between the sacrotuberous ligament and the sciatic nerve. However, this was beyond the scope of this study. Further studies should be performed in different dogs breeds with different height ranges to investigate the spatial relationship of the sacrotuberous ligament and the sciatic nerve in those breeds.
